# Supplementary material for: Metatranscriptomic Analysis Reveals an Imbalance of Hepatopancreatic Flora of Chinese Mitten Crab Eriocheir sinensis with Hepatopancreatic Necrosis Disease
Source: Biology (Basel). 2021 May 23;10(6):462. doi: 10.3390/biology10060462 (PMC8224665; doi:10.3390/biology10060462)
Supplement: Supplementary file 1 [file biology-10-00462-s001.zip › biology-1205556-supplementary.pdf]

Biology
Supplementary data

Hepatopancreatic Necrosis Disease of Chinese Mitten Crab Eriocheir sinensis not caused by Virus or Microsporidia Infection

Zeen Shen[1], Dhiraj Kumar[1,4], Xunmeng Liu[2], Bingyu Yan[1], Ping Fang[2], Yuchao Gu[1], Manyun Li[1], Meiping Xie[1], Rui Yuan[2], Yongjie Feng[1], Xiaolong Hu[1,3], Guangli Cao[1,3], Renyu Xue[1,3], Hui Chen[2], Xiaohan Liu[2], Chengliang Gong[*1,3]

[1] School of Biology and Basic Medical Science, Soochow University, Suzhou 215123, China
[2] Jiangsu Center for Control and Prevention of Aquatic Animal Infectious Disease, Nanjing 210036, China
[3] Agricultural Biotechnology Research Institute, Agricultural Biotechnology and Ecological Research Institute, Soochow University, Suzhou, 215123, China
[4] School of Studies in Zoology, Jiwaji University, Gwalior-474011, India
* Correspondence: gongcl@suda.edu.cn

Figure S1 3
Figure S2 3
Figure S3 10
Figure S4 11
Figure S5 11
Figure S6 12
Figure S7 12
Table S1 13
Table S2 13

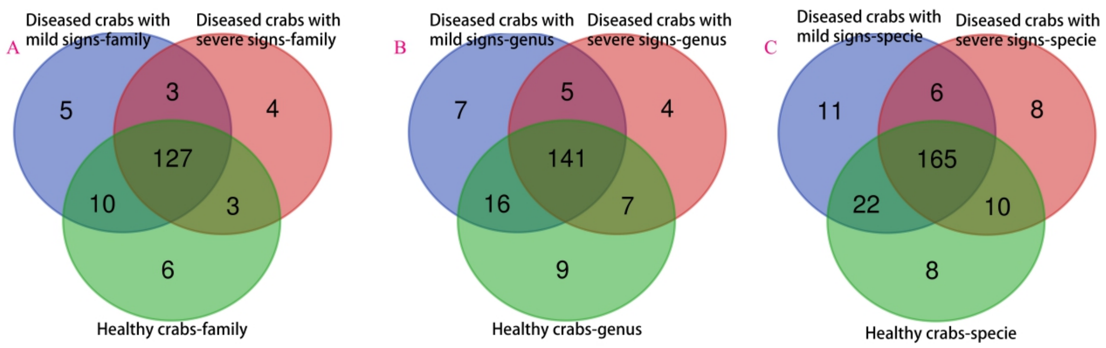

Figure S1 Venn diagram of species identified by metatranscriptomic sequencing in different samples at different taxonomic levels

A, B and C represent venn diagram at the family, genus and specie levels.

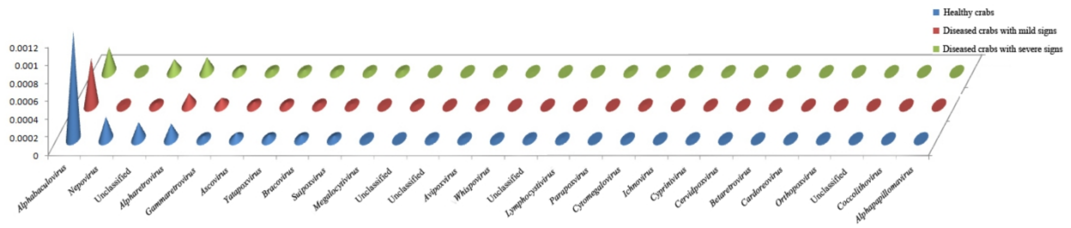

Figure S2 Relative abundance of viral genera among different samples

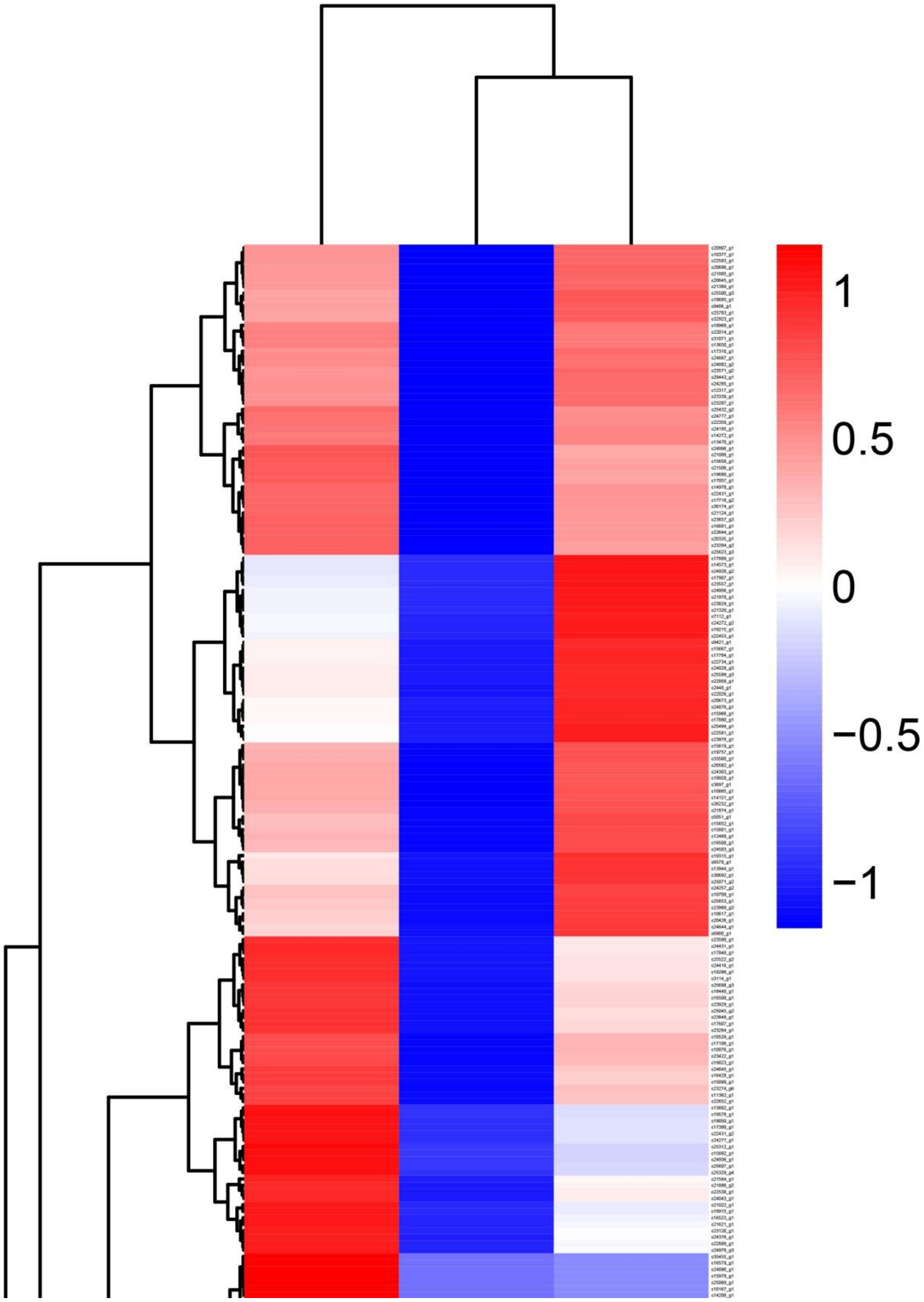
1
0.5
0
−0.5
−1

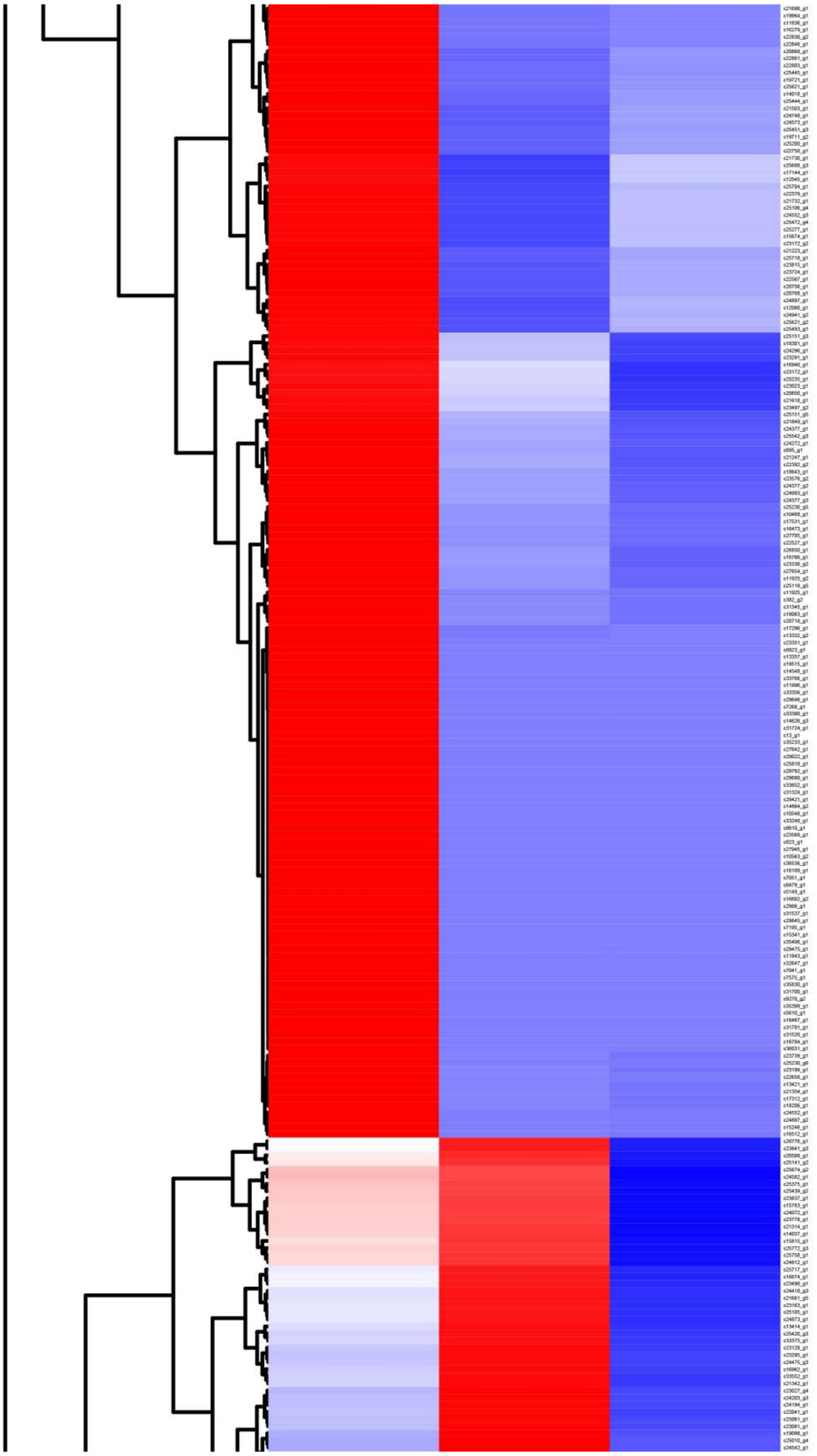

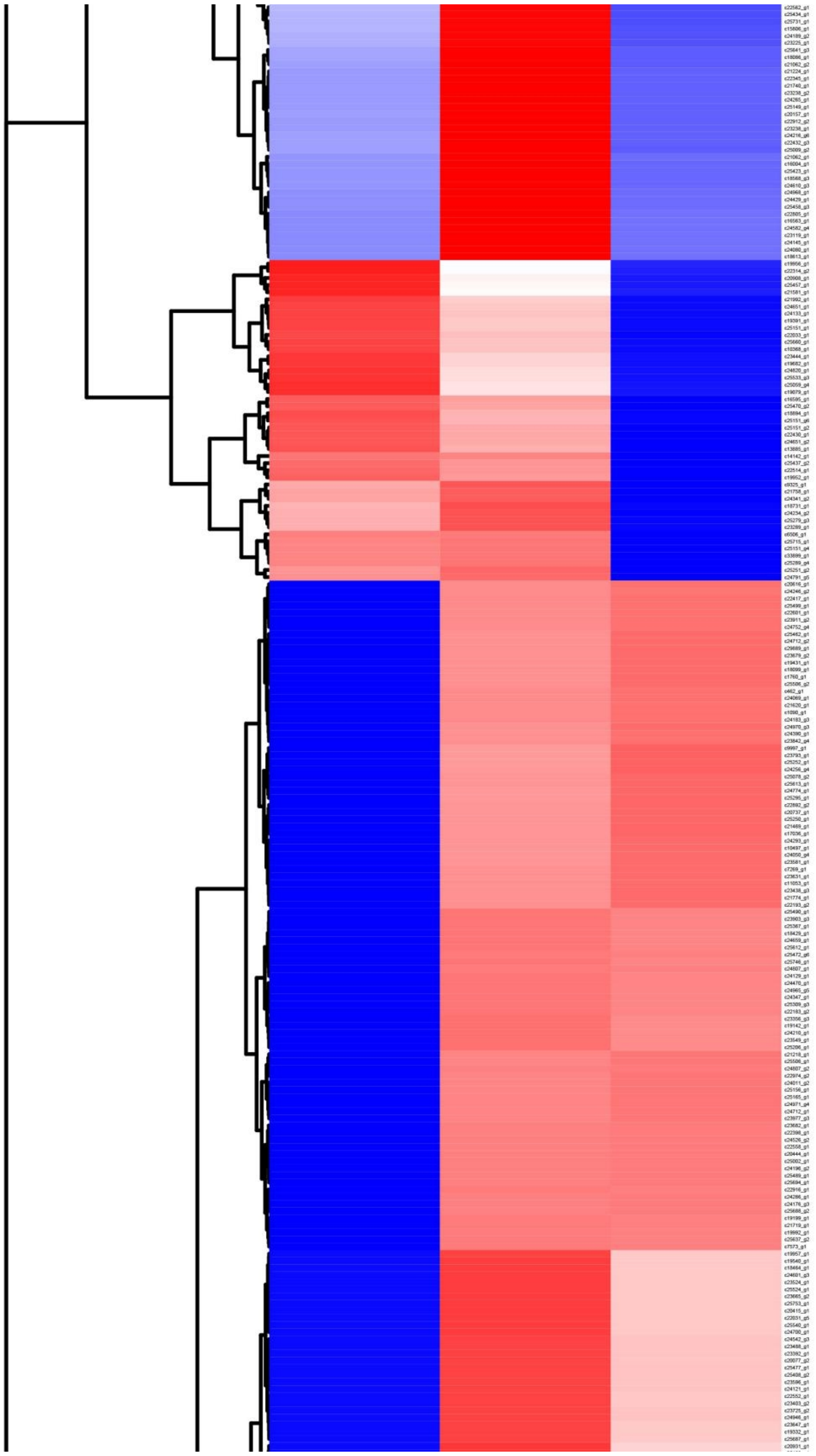

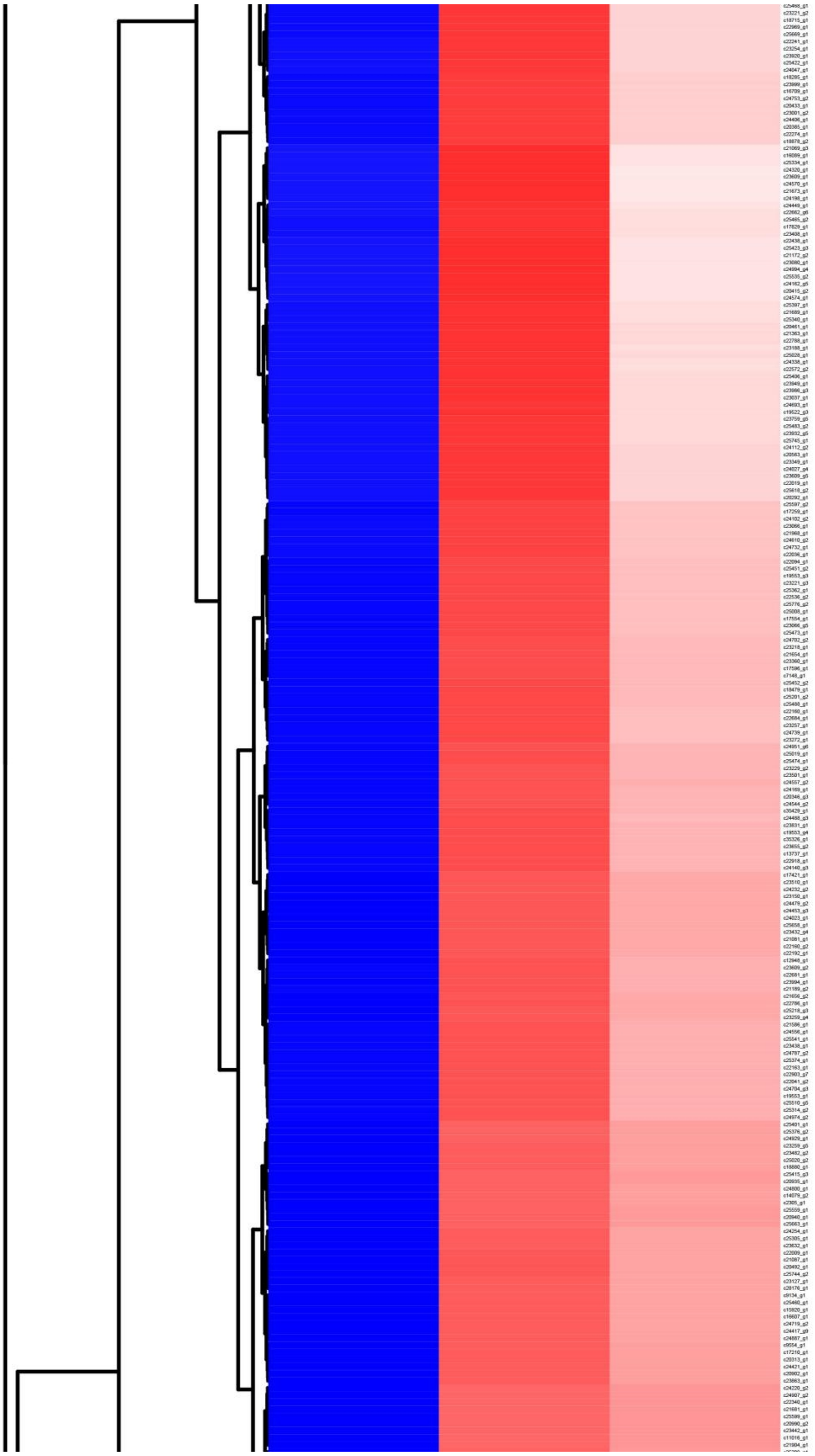

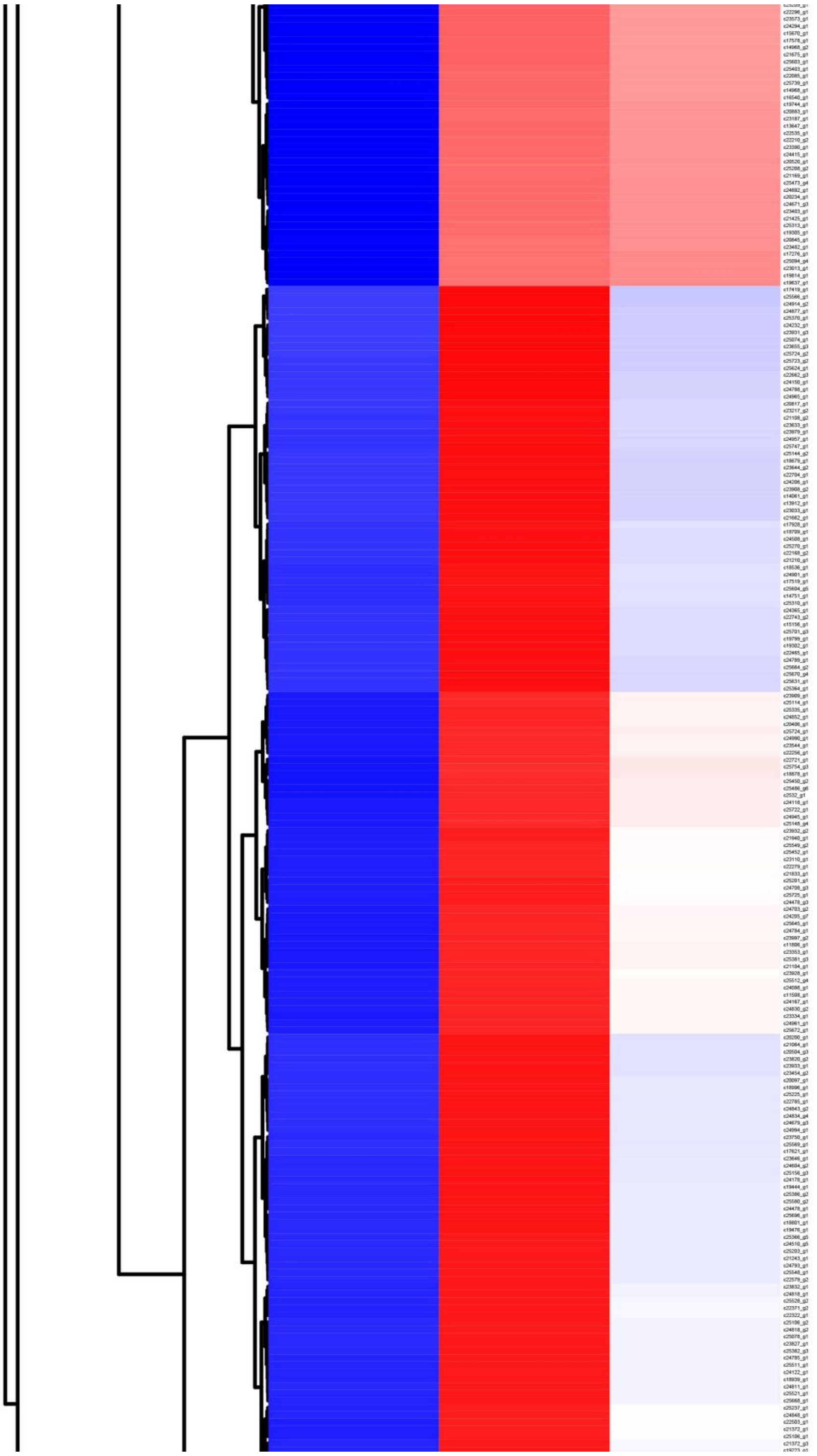

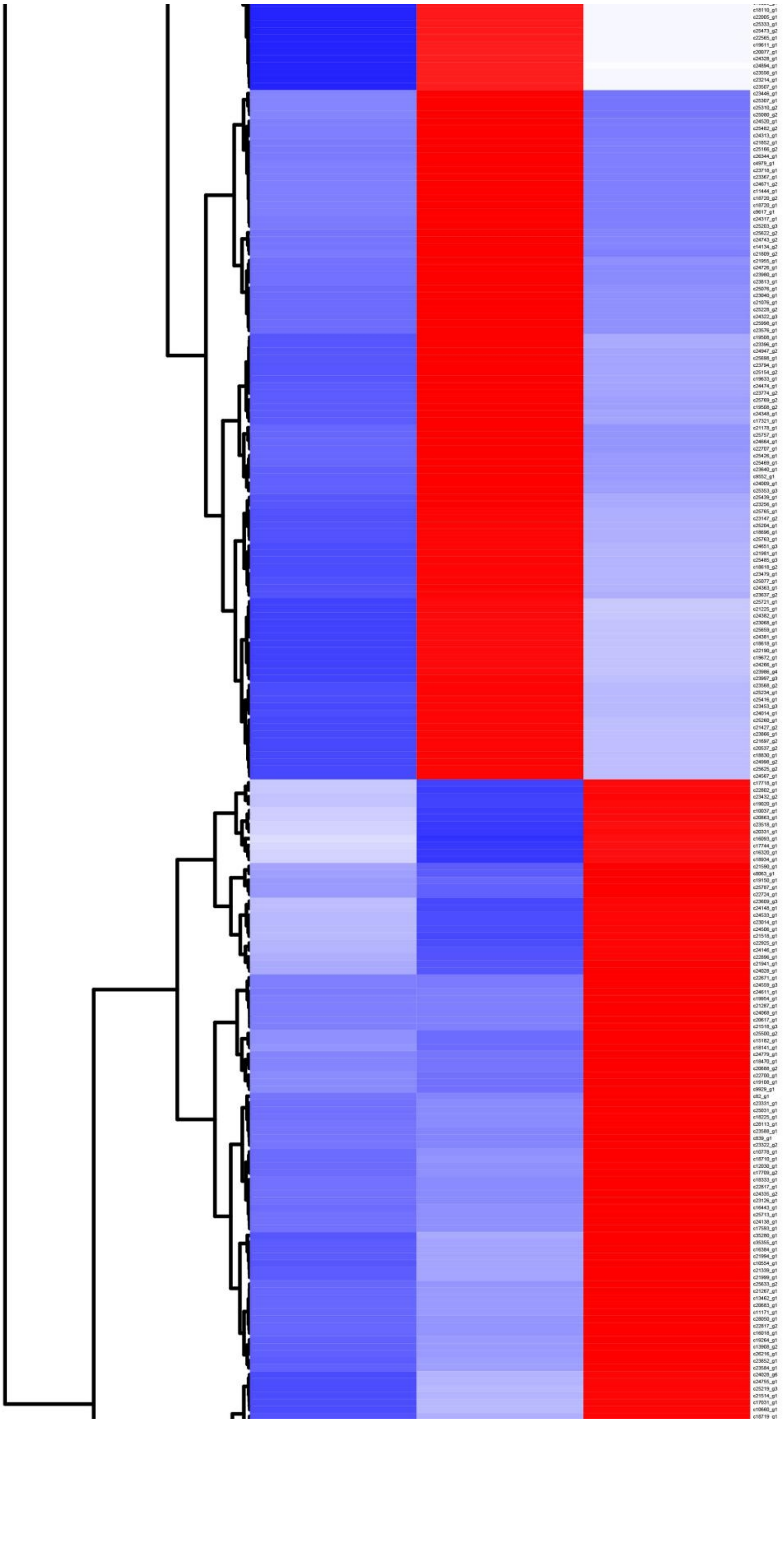

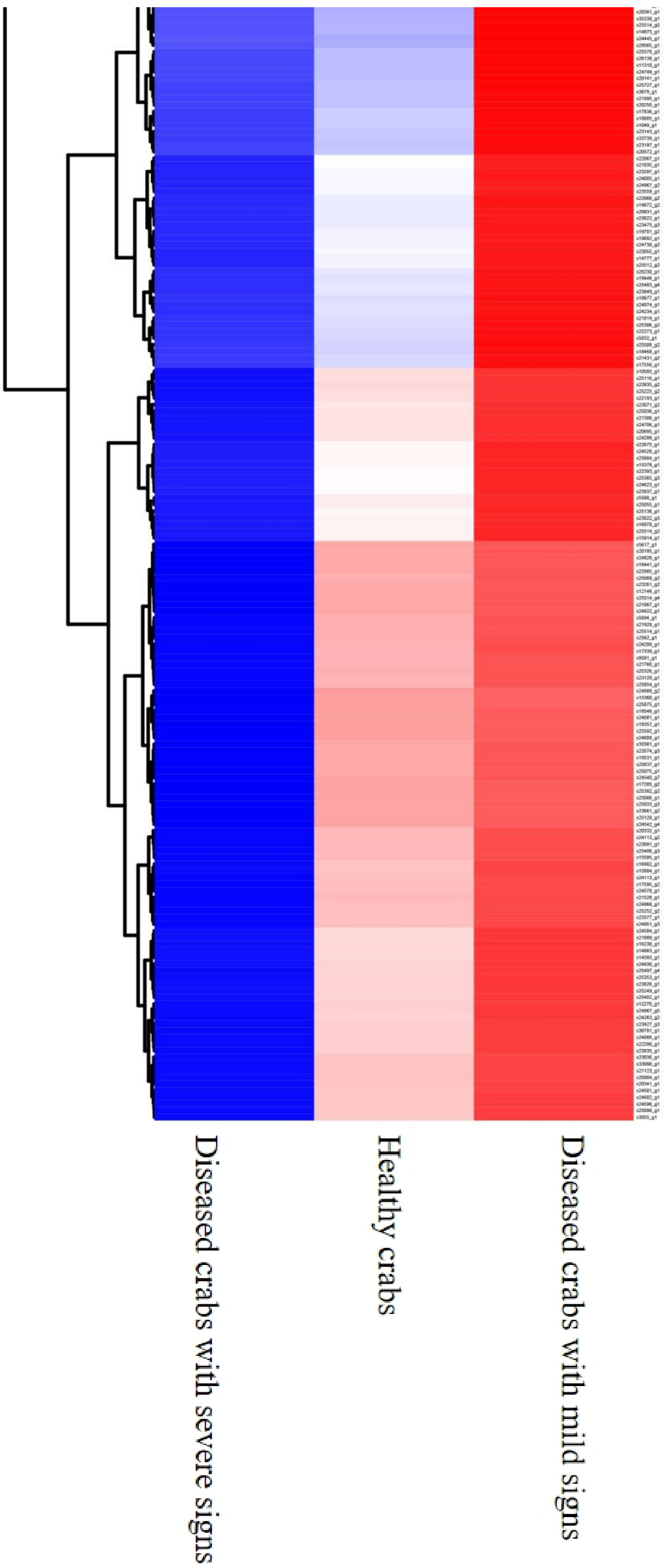

Figure S3 Heatmap of all DEGs among Diseased crabs with mild signs and

Healthy crabs, Diseased crabs with severe signs and Healthy crabs, and Diseased crabs with severe signs and Diseased crabs with mild signs

DEGs were identified by metatranscriptomic sequencing, the colour intensity represents relative expression level, which is named as z-value and generated by the relative expression level of a gene in each line after normalization treatment.

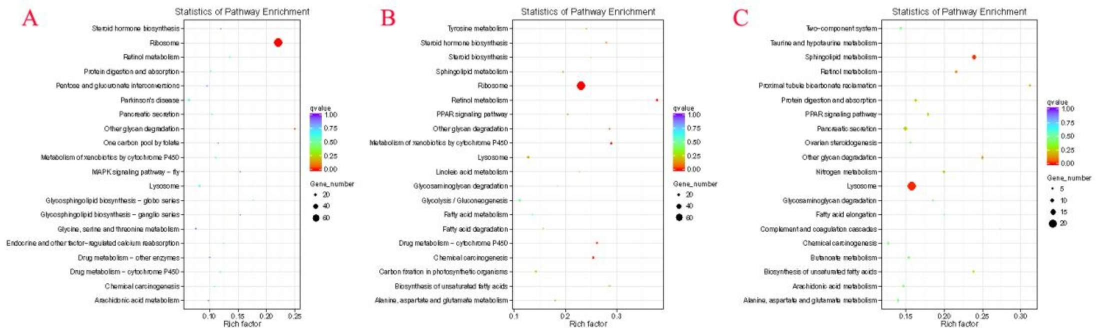

Figure S4 Top 20 enriched KEGG pathways of DEGs identified in the hepatopancreatic flora

A, Diseased crabs with mild signs *vs.* Healthy crabs; B, Diseased crabs with severe signs *vs.* Healthy crabs; Diseased crabs with severe signs *vs.* Diseased crabs with mild signs

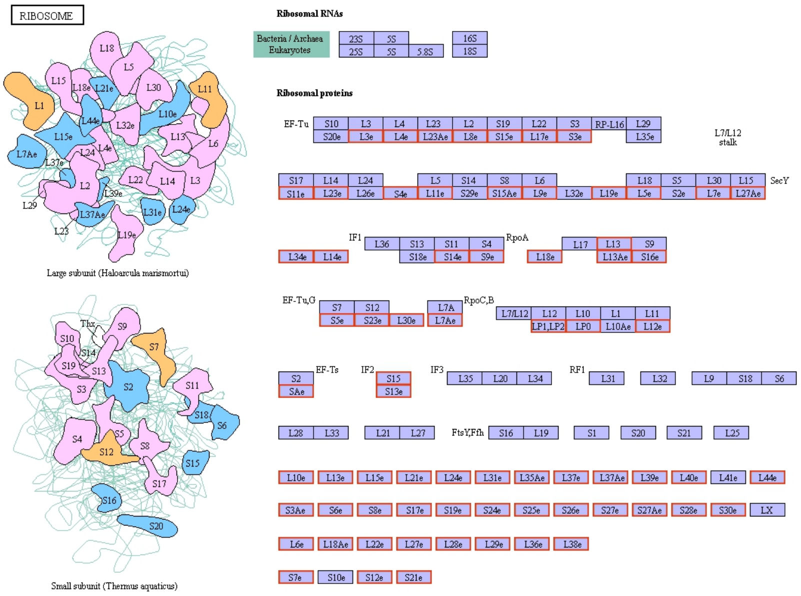

Figure S5 71 enriched genes to ribosomes in diseased crabs with severe signs vs. healthy crabs. The red boxes represent the upregulated genes.

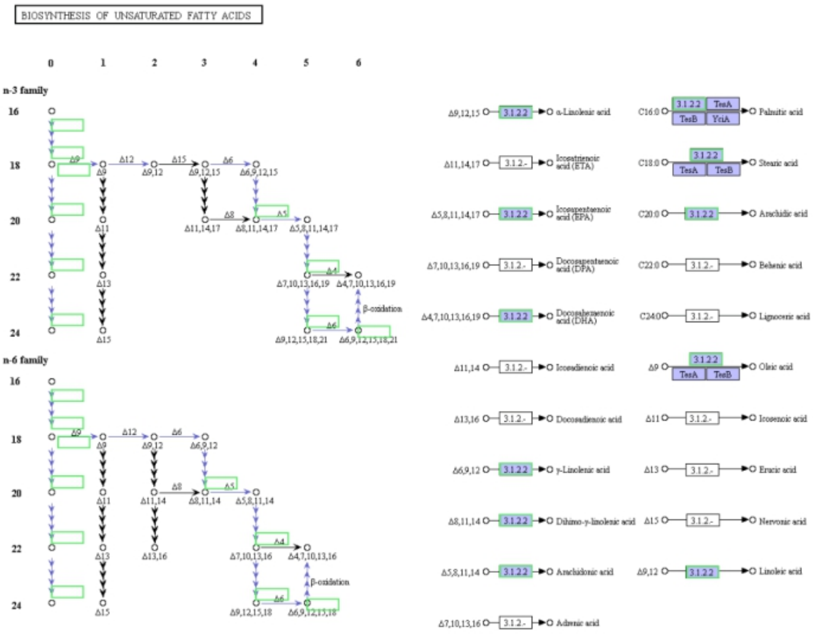

Figure S6 Enriched genes to the biosynthesis of unsaturated fatty acids in diseased crabs with severe signs vs. healthy crabs was downregulated. The green boxes represent the downregulated genes.

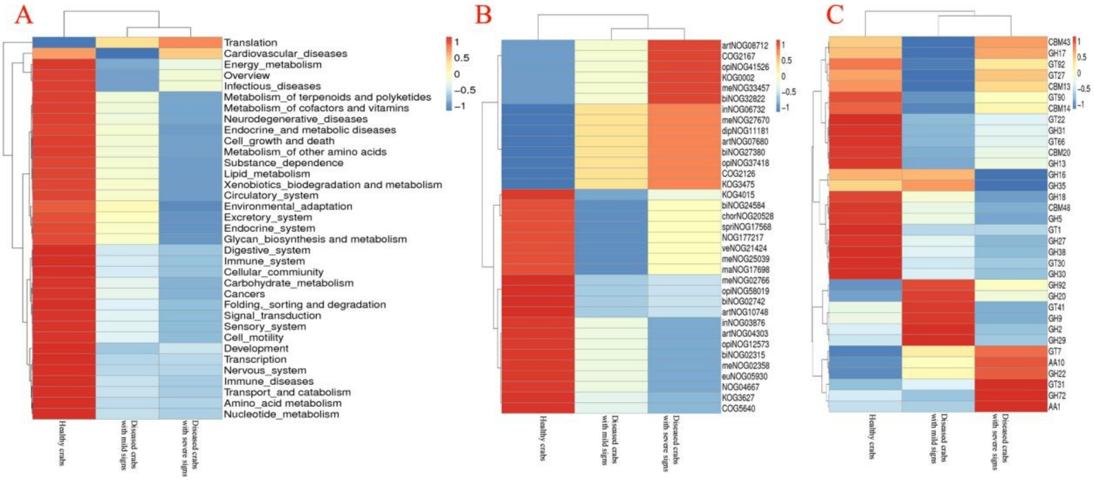

Figure S7 Heatmap of KEGG, eggNOG and CaZy between samples

A, KEGG; B, eggNOG; C, CaZy. The horizontal axis represents sample information; the vertical axis represents species information; the cluster tree on the left side functions clustering tree; the colour intensity in the square grid represents the functional abundance, which is named as z-value and generated by the relative abundance of function in each line after normalization treatment.

Table S1 Primers used in this study

Primers	sequences	PCR product (Kb)	Assigned viruses
CSBV-1	GGATCTGGGGTCGATCAGTAC	0.3	*Cotesia sesamiae bracovirus*
CSBV-2	GTTCCTCCTAACTGCTTGTCC		
NV-hel-1	GGGTCTGTTATGGTACCAGGA	0.25	*Penaeus monodon nudivirus*
NV-hel-2	ATCTTCCCTGGTGAATTTACC		
Reo-905-1	GGCTCGATATGCTCAGAACG	0.6	*Eriocheir sinensis reovirus*
Reo-905-2	CTTGCGATCAGGCAGACCAG		
MDWSSV-1	CCTACACAGTCCGTCGTGAAG	0.2	*Metopaulias depressus WSSV-like virus*
MDWSSV-2	CGTTCCGTTGACCATAGGGAG		
SPI-1	ATGAAGAAGTTAATAGCG	0.84	*Spiroplasma eriocheiris*
SPI-2	GCGTCGACTTACTTTACACTG GGAAGCAC		

Table S2 Data quality of metatranscriptomic sequencing

Sample	Raw Reads	Clean Reads	Clean Bases	Error(%)	Q20(%)	Q30(%)	GC Content (%)
Healthy crabs	34,431,454	32,807,952	4.92 G	0.01	97.86	94.22	54.75
Diseased crabs with mild signs	30,929,290	29,532,670	4.43 G	0.01	97.85	94.26	54.36
Diseased crabs with severe signs	29,709,614	27,582,606	4.14 G	0.01	98.08	95	55.93
